# Laparoscopic adrenalectomy for a giant adrenal myelolipoma: A case report^☆^

**DOI:** 10.1016/j.ijscr.2021.106678

**Published:** 2021-12-11

**Authors:** F.P. Tinozzi, G. Morone, B. Calì, A. Rebba, N. Osman, S. Albertario, F. Abbiati, R. Ruggiero

**Affiliations:** a Department of General and Mininvasive Surgery, ICS Maugeri IRCCS, Pavia, Italy; b Occupational Medicine Unit, ICS Maugeri IRCCS, Pavia, Italy; c Occupational Medicine Unit, Department of Public Health, Experimental and Forensic Sciences, University of Pavia, Italy

**Keywords:** Laparoscopic surgery, Adrenal glands, Giant myelolipoma

## Abstract

**Case presentation:**

We describe a case of a patient who presented with a mildly symptomatic, giant myelolipoma which was excised by laparoscopic approach without complications.

**Introduction and importance:**

Adrenal myelolipoma (AML) is a rare tumour composed by fat and myeloid tissues. Usually it is asymptomatic, so the diagnosis is mostly incidental. It is generally located in the right adrenal gland, but it can also be found bilaterally. If its size exceeds 10 cm it is defined as a “giant myelolipoma”; in this case its treatment of choice would be adrenalectomy with an open surgical approach.

**Clinical discussion:**

Patient's signs and symptoms were mild pain in the right hypochondrium and a positive right Giordano's sign. The mass was detected by a contrast-enhanced CT scan. Once excised it measured 16 cm.

**Conclusion:**

Laparoscopic adrenalectomy for giant myelolipoma is a safe approach if performed by an expert surgeon, with low risk of bleeding and a better outcome for the patient.

## Highlights

• Literature evidences are scarce regarding the surgical management of giant adrenal myelolipomas.
• Laparoscopic surgery is safe and feasible even for adrenal lesions larger than 10 cm.
• Clear resection margins can be obtained even for massive adrenal tumors.

## 1 Introduction

Adrenal myelolipoma (AML) is a non-functioning benign mesenchymal tumour composed of mature adipose and myeloid tissue. Both sexes are equally affected by AML, mainly in the fifth-sixth decade of life. It is a rare tumour with an incidence of 0.08–0.2% at autopsy [1].

It is frequently diagnosed incidentally, given its asymptomatic nature. Its diagnosis has become simpler and more frequent thanks to the widespread use of imaging studies like Ultrasonography (US), computed tomography (CT), and Magnetic Resonance Imaging (MRI). If its diagnosis cannot be certified with imaging, and hence malignancy suspicion remains, a fine needle biopsy should be performed [2]. On average its size is smaller than 4 cm. If it exceeds 10 cm, it is defined as “giant myelolipoma” [3].

Adrenal myelolipomas were first described by Gierke in 1905 [4] and first referred as myelolipoma by Oberling in 1929 [5].

AML is a rare tumour [6], which affects equally both sexes, mainly in the fifth-sixth decade of life. Their incidence is about 1% in the younger populations (under 30 years) and 7% in the elderlies (over 70 years) [7].

AML is a benign mesenchymal tumour with different cell types, mainly adipocytes and haematopoietic cells, which include erythroid and myeloid elements. They are not extramedullary sites of haematopoiesis [8]. Usually an endocrinological examination (plasma epinephrine, norepinephrine, metanephrine, renin, ACTH, cortisol, aldosterone, 17-OH-progesterone, 24 h urinary cortisol, free cortisol, vanillymandelic acid and homovanillic acid) is evaluated to detect whether an endocrinal disorder or a secreting neoplasm is present [9]. In our case, we didn't find any endocrinological alterations.

Most commonly myelolipomas are found in the right adrenal gland, but it can also be found bilaterally. Sometimes they were described in the presacral area, stomach, spleen, liver, lung and testes [10].

Rarely it presents with unspecific abdominal symptoms like pain, abdominal discomfort, nausea, vomiting, weight loss, diarrhea, and dyspepsia, most of them for its mass effects, impinging on the surrounding organs; some patients experience episodes of haematuria or signs of internal haemorrhage. As they grow larger than 10 cm in diameter, they could rupture spontaneously. Although, according to Meyer et al. there isn't correlation between symptoms and the dimension of the tumour [11]. Because it often presents with no evident symptoms, the diagnosis is frequently incidental, in fact this kind of tumour is often discovered during imaging investigations for other causes or during autopsy [12].

Recently, the widespread use of imaging studies increased the detection of AML [13]. CT scan is the most sensitive diagnostic imaging modality: myelolipomas are described as well delineated heterogeneous masses with low-density mature fat (less than −30 Hounsfield Units [HU]) among more dense myeloid tissue; they can be encapsulated, and the soft tissue component is enhanced after the administration of intravenous contrast [14]. In our case ultrasonography and CT scan were suggestive for AML.

On MRI, instead, the fatty component is usually hyperintense on the T1-weighted images and heterogeneously hyperintense on the T2-weighted images [15].

However, MRI doesn't help in carrying out the diagnosis of adrenal myelolipoma in patients with unclear CT findings, and, thereof, needle biopsy could be helpful in such difficult cases [16].

Conservative approach is usually preferred as treatment strategy. However, if symptomatology ensues, mass growth increases speedily, or if presents as greater than 6 cm, adrenalectomy will be the main indication. In fact, the potential risk of malignancy and retroperitoneal haemorrhage would be substantial.

Thereby, the mainstay of surgical treatment of giant myelolipomas is radical adrenalectomy through an open surgical approach [17]. In this case report we describe the surgical treatment of a silent giant AML of 16 cm through laparoscopic surgery.

## 2 Case report

A 61-year-old Caucasian male was admitted to our General Surgery Department for a two-month persistent pain in the right hypochondrium, with no other relevant symptomatology. His medical history included hypertension, obesity (BMI 30,24 kg/m2), aortic valve replacement with bio-prosthesis performed in 2018 for severe aortic valve stenosis.

Preoperative abdominal US revealed a mass bigger than 10 cm with a heterogeneous appearance. It appeared mainly hyperechoic with hypoechoic islets, interposed between the lower hepatic margin and the upper renal pole. Vascularization at colour-Doppler sonography was non-significant.

**Fig. 1:**
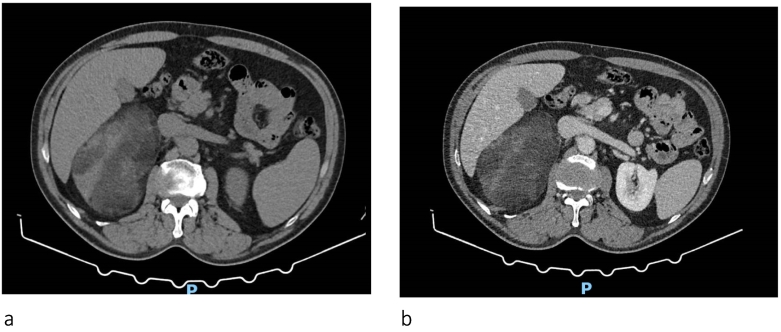
CT scan with basal acquisition (a) and with contrast (b). Voluminous, solid lesion with poor acquisition of contrast and well-defined, regular contours.

A contrast-enhanced abdominal CT scan confirmed the presence of a voluminous (14 × 12 cm) encapsulated neoformation composed of mainly adipose tissue in the right adrenal lodge. It arose from the medial branch of the right adrenal gland, in contiguity with the upper third of the right kidney, which appeared dislocated and partially horizontalized on its major axis (Fig. 1a, b).

**Fig. 2:**
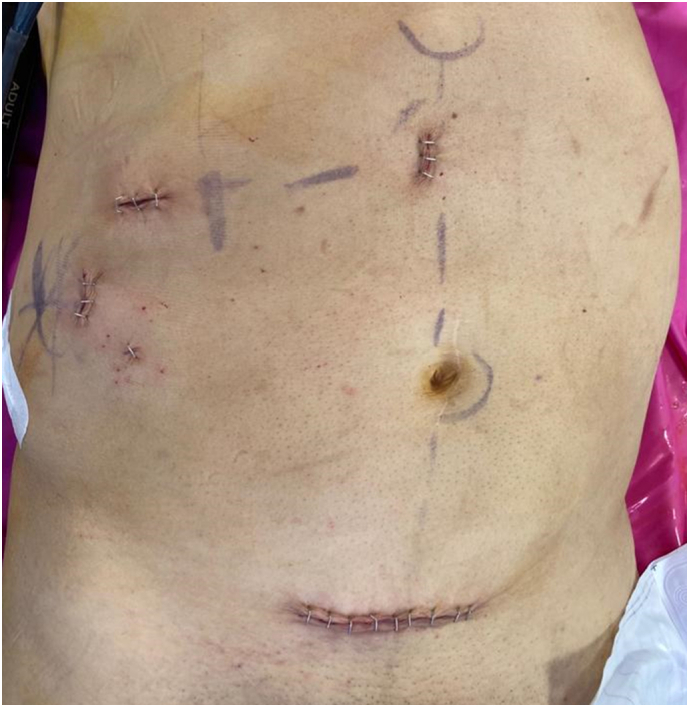
Port sites after the surgical operation.

Laboratory data showed normal hormonal levels: aldosterone 40.60 pg/ml [37–432 pg/ml]; serum cortisol 10.1 μg/dL [3.7–19.4 μg/dl], adrenocorticotropic hormone 17.60 pg/mL [≤46 pg/ml]; dehydroepiandrosterone sulfate 91.90 μg/dl [48.6–361.8 μg/dl]; renin 10.10 microUI/ml [5.3–99.1 microUI/ml]; 24-h urinary metanephrines 111.84 microg/24 h [0–374.99] and normetanephrines 326.77 microg/24 h 0–779.99. Dexamethasone suppression test revealed adequate decrease of serum cortisol <1 μg/dL. Abdominal physical examination was unremarkable. All radiological features showed no signs of malignancies. However, due to patient's discomfort a laparoscopic procedure was performed. Under general anaesthesia, an anterior transabdominal approach using four trocars was carried out. Our patient was placed in a left flank position with a 45 ° side tilt. The optical trocar (11 mm) was inserted on the intersection between the trans-umbilical and periumbilical lines. The liver retractor trocar (11 mm) was placed subxiphoidally on the paraumbilical line. The last two trocars (11 mm and 5 mm) were positioned at the level of the mammary line, respectively in the subcostal and right iliac regions. During dissection, the tumour had a red-brown pigmentation, due to the presence of focal areas of haemorrhage. After right adrenalectomy, the specimen was placed in an Endopouch inserted through a Pfannenstiel incision (Fig. 2).

**Fig. 3:**
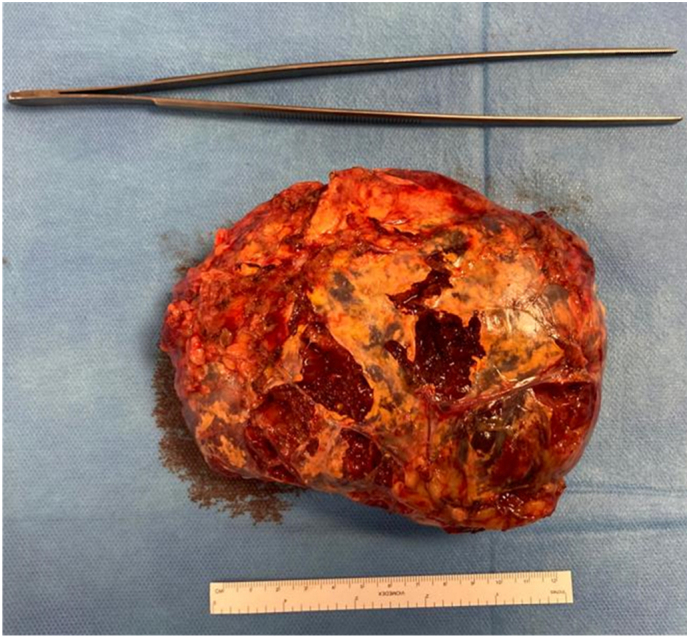
Surgical specimen extracted through a Pfannenstiel incision.

Histological examination revealed a large encapsulated adrenal myelolipoma measuring 16 × 12 × 6 cm and weighing 634 g (Fig. 3).

The operative time (OT) was of 160 min and no clinical intra-or postoperative complications occurred. Laboratory data after surgery didn't present any alteration and no laboratory signs of post-operative complications were observed.

On the 1st postoperative day (POD) the patient was mobilized. Oral feeding was initiated 2 days after surgery and on the 4th POD the abdominal drain was removed. The patient was discharged in good general condition on the 5th postoperative day. At 6 month-follow up no signs of recurrence were detectable.

## 3 Discussion

Treatment for adrenal masses is required for lesions with the following features: clinically relevant, hormonally active, or large in size. The risk of malignancy for adrenal masses increases to 25% in lesions bigger than 6 cm, therefore this is the surgical cut-off value. Small, non-functioning tumors should be managed via annual radiological follow up while the ones greater than 7 cm should be removed. Instead, giant myelolipomas (greater than 10 cm) are even more commonly associated with intraoperative complications, such as bleeding, capsular breach and a higher risk of local recurrence [18].

Open radical adrenalectomy is the standard treatment for giant myelolipomas or in emergency cases wherein haemorrhage or rupture occur, while the minimally invasive approach is preferred for small AML [19]. In the literature it is reported that the largest myelolipoma ever excised measured 31 × 24.5 × 11.5 cm and weighted 6 kg. The surgical procedure was performed by Akamatsu in open surgery [20].

The first laparoscopic adrenalectomy was described by Gagner in 1992 [21]. There are different advantages of the minimally invasive approach compared with the traditional approach: less pain, shorter hospitalization, and faster recovery [22].

Only few cases of giant myelolipomas were treated with minimally invasive adrenalectomy [23]. Our case proves that giant AML could be removed safely with laparoscopic approach, without any intra- or post-operative complication and providing a better outcome to the patient.

## 4 Conclusion

Very scarce evidence can be found in the literature regarding the laparoscopic surgical approach of giant adrenal masses. The gold standard yet remains the open surgical approach, however thanks to the improvements of minimally invasive techniques, we have presented a case wherein this approach can be considered safe and feasible. Furthermore, clear resection margins and a 6-month negative follow up can be achieved.

## Sources of funding

No source of funding.

## Ethical approval

Not applicable.

## Consent

Written informed consent was obtained from the patient for this case report and accompanying images. A copy of the written consent is available for review by the Editor-in-Chief of this journal on request.

## Provenance and peer review

Not commissioned, externally peer-reviewed.

## Guarantor

Dr. Tinozzi Francesco Paolo, MD.

## Registration of research studies

Not applicable.

## CRediT authorship contribution statement

Dr. Tinozzi Francesco Paolo, MD Study design, study concept, data collection, final approval of the version to be submitted, surgical operation

Dr. Morone Giovanni, MD Study design, study concept, data collection, data analysi, surgical operation

Dr. Benedetto Calì, MD, Study design, study concept, data collection, data analysis, writing the article

Dr. Rebba Andrea, MD, Study design, study concept, data collection, data analysis, writing the article

Dr. Osman Nadine, MD, study concept, data collection, data analysis

Dr. Albertario Simone, MD, study concept, data collection, data analysis

Dr. Abbiati Francesca, MD, study concept, data collection, data analysis

Dr. Ruggiero Rubina, MD, Study design, study concept, data collection, surgical operation

## Declaration of competing interest

No conflict of interest is present.
